# Mapping a Novel Black Spot Resistance Locus in the Climbing Rose Brite Eyes™ (‘RADbrite’)

**DOI:** 10.3389/fpls.2018.01730

**Published:** 2018-11-26

**Authors:** Jason D. Zurn, David C. Zlesak, Matthew Holen, James M. Bradeen, Stan C. Hokanson, Nahla V. Bassil

**Affiliations:** ^1^USDA-ARS National Clonal Germplasm Repository, Corvallis, OR, United States; ^2^Department of Plant and Earth Science, University of Wisconsin River Falls, River Falls, WI, United States; ^3^Department of Horticulture, University of Minnesota, St. Paul, MN, United States; ^4^Department of Plant Pathology, University of Minnesota, St. Paul, MN, United States

**Keywords:** RosBREED, host-resistance, polyploid, ornamental plants, SNP

## Abstract

Rose black spot, caused by *Diplocarpon rosae*, is one of the most devastating foliar diseases of cultivated roses (*Rosa* spp.). The globally distributed pathogen has the potential to cause large economic losses in the outdoor cultivation of roses. Fungicides are the primary method to manage the disease, but are often viewed unfavorably by home gardeners due to potential environmental and health impacts. As such, rose cultivars with genetic resistance to black spot are highly desired. The tetraploid climbing rose Brite Eyes^TM^ (‘RADbrite’) is known for its resistance to black spot. To better characterize the resistance present in Brite Eyes^TM^, phenotyping was conducted on a 94 individual F_1_ population developed by crossing Brite Eyes^TM^ to the susceptible tetraploid rose ‘Morden Blush’. Brite Eyes^TM^ was resistant to all *D. rosae* races evaluated except for race 12. The progeny were either resistant or susceptible to all races (2, 3, 8, 9, 10, 11, and 13) evaluated. The segregation ratio was 1:1 (χ^2^ = 0.3830, *P* = 0.5360) suggesting resistance is conferred by a single locus. The roses were genotyped with the WagRhSNP 68K Axiom array and the ‘polymapR’ package was used to construct a map. A single resistance locus (*Rdr4*) was identified on the long arm of chromosome 5 homoeolog 4. Three resistance loci have been previously identified (*Rdr1, Rdr2*, and *Rdr3*). Both *Rdr1* and *Rdr2* are located on a chromosome 1 homoeolog. The chromosomal location of *Rdr3* is unknown, however, races 3 and 9 are virulent on *Rdr3*. *Rdr4* is either a novel gene or an allele of *Rdr3* as it provides resistance to races 3 and 9. Due to its broad resistance, *Rdr4* is an excellent gene to introgress into new rose cultivars.

## Introduction

Black spot of rose, caused by *Diplocarpon rosae* Wolf, is one of the most devastating foliar diseases impacting outdoor grown roses, *Rosa* spp. L. The black spot pathogen is globally distributed and most modern cultivars are susceptible to at least one of the 13 reported races (Gachomo et al., 2006; Whitaker et al., 2010b). Roses are among the most important ornamental crops with global production valued at an estimated $28 billion, $1 billion of which is due to production in North America (Debener and Byrne, 2014). Approximately 70 million potted roses and 220 million roses for landscaping are sold each year (Debener and Byrne, 2014). As such, the pathogen is poised to cause large economic losses.

*Diplocarpon rosae* has a hemibiotrophic life cycle, making it unique among the majority of plant pathogenic fungi (Gachomo et al., 2006). During the infection process, the spore germinates and develops a germ tube and appressorium to penetrate the leaf cuticle (Gachomo et al., 2006). A subcuticular vesicle is formed and intercellular hyphae grow and form haustoria (Gachomo et al., 2006). These haustoria and intercellular hyphae produce biotrophic effectors that the pathogen uses to circumvent the host defense response. At approximately 6 days after inoculation on susceptible germplasm, the pathogen switches from a biotrophic life style to a necrotrophic life style and forms necrotrophic intracellular hyphae (Gachomo et al., 2006) that release necrotrophic effectors designed to kill the plant tissue. Shortly after this change, an acervulus and conidia are formed which can become viable as early as 9 days after inoculation (Gachomo et al., 2006). The conidia are then spread by water to start the infection process on new leaves.

Black spot has traditionally been managed with fungicide applications either solely or as part of an integrated pest management system. Multiple applications of fungicides are required annually to control the disease, due to the macrocyclic life cycle of the pathogen. To the average home gardener and public garden managers, multiple fungicide applications are viewed unfavorably due to the risk of chemical exposure, as well as the added cost and labor associated with multiple applications (Harp et al., 2009). An increased demand has arisen for “low-maintenance” rose cultivars that require low water and fertilizer input and are disease and pest resistant, tolerant of abiotic stresses, and esthetically pleasing (Zlesak, 2006; Harp et al., 2009; Byrne, 2015; Waliczek et al., 2015).

Genetic resistance is a critical component to managing diseases in an environmentally friendly and cost-effective manner. Very little is known about loci associated with black spot resistance and only three resistance loci (*Rdr1, Rdr2*, and *Rdr3*) have been identified (von Malek and Debener, 1998; Hattendorf et al., 2004; Whitaker et al., 2010a; Menz et al., 2017). Partial resistance to rose black spot has also been observed (Xue and Davidson, 1998; Whitaker and Hokanson, 2009; Dong et al., 2017). The rose Brite Eyes^TM^ (‘RADbrite’), exhibited resistance to three races (3, 8, and 9) tested during a black spot resistance trial of 75 rose cultivars (Zlesak et al., 2010). To better characterize the resistance present in Brite Eyes^TM^ and identify loci associated with black spot resistance, a mapping population was developed and evaluated with multiple *D. rosae* races and the WagRhSNP 68K Axiom SNP array (Koning-Boucoiran et al., 2015).

## Materials and Methods

### Population Development and Phenotypic Evaluation

A 94 individual F_1_ two-way pseudo test cross mapping population was developed at the University of Minnesota by crossing Brite Eyes^TM^ to the female parent ‘Morden Blush’. Both Brite Eyes^TM^ and ‘Morden Blush’ are tetraploid (2*n* = 4*x* = 28; Zlesak et al., 2010) and the resulting offspring were expected to be tetraploid. The plants were maintained in containers in the greenhouse using a peatlite blend with bark and ∼20% pasteurized loam soil added to buffer pH and help provide micronutrients. Plants were transplanted as needed and nutrition was provided using different water soluble fertilizers at appropriate concentrations and frequencies depending on the time of the year and how actively the plants were growing (i.e., less during the winter months). A sulfur burner was used for 5 h nightly to prevent the growth of powdery mildew.

Brite Eyes^TM^ and ‘Morden Blush’ were evaluated with 13 *D. rosae* isolates from North America and Europe each of which represented a different race to determine the breadth of the resistance in Brite Eyes^TM^ (Table 1). Isolates were maintained on susceptible leaf tissue prior to use and phenotyping was conducted at the University of Minnesota using a detached leaf assay. For each plant, two leaves, each comprised of 3 to 7 leaflets, were washed by dipping in 70% isopropanol and then rinsing three times with ddH_2_O. The leaves were blotted dry and placed in a container with a moist paper towel. After blotting, a pipette was used to inoculate the leaves by dispensing 750 μL of a 3.0–8.0 × 10^4^ spores/mL conidiospore suspension in autoclaved distilled water on each leaf. The leaves were incubated for 14 days in the laboratory in clear plastic containers with a sheet of moist paper towel to keep the humidity high. The inoculations occurred in the laboratory where the temperature was held constant at approximately 22°C and a 24 h photoperiod was maintained with the cool white overhead florescent lights to help keep the leaves green and slow down leaf senescence. Droplets on the surface of the leaves from inoculation were blotted from the leaf surface at 2 days post inoculation to minimize secondary infections. At the end of the incubation period, susceptible genotypes were identified by looking for expanding black spot lesions. Questionable reactions were confirmed as susceptible by looking for the presence of acervuli at 40 × magnification with a dissection microscope. Resistant genotypes did not have any visible lesions or only had small lesions without acervuli that did not expand from the inoculation point. The 94 individuals in the population were evaluated as previously described with seven isolates (races 2, 3, 8, 9, 10, 11, and 13) that displayed a differential reaction between the parents.

**Table 1:** Phenotypes of Brite Eyes^TM^ and ‘Morden Blush’ to 13 *D. rosae* isolates from North America and Europe. Phenotypes are recorded as resistant (-) or susceptible (+).

Isolate	Race	Origin	Brite Eyes^TM^	‘Morden Blush’
HSN	1	North America	-	+
2402 E1	2	Germany	-	+
GVH	3	North America	-	+
DüA3	4	Germany	-	+
B005	5	Belgium	-	+
DortE4	6	Germany	-	+
R6	7	Germany	-	+
ACT	8	North America	-	+
IGWA	9	North America	-	+
KOMN	10	North America	-	+
CW1	11	United Kingdom	-	+
BEP	12	North America	+	+
PAP	13	North America	-	+

### Genotyping and Linkage Mapping

Actively growing leaf tissue was collected from the parents and offspring growing in a greenhouse at the University of Minnesota and mailed overnight to the USDA-ARS National Clonal Germplasm Repository (NCGR). Tissue was sampled into a 96 well plate and DNA was extracted using the E-Z 96 Plant DNA extraction kit (Omega BioTek, Norcross, GA, United States) as previously described (Gilmore et al., 2011). The Quant-iT Picogreen Assay (Invitrogen, Eugene, OR, United States) and a Victor^3^V 1420 Multilabel Counter (Perkin Elmer, Downers Grove, IL, United States) were used to quantify the extracted genomic DNA. The DNA concentration of each sample was adjusted to 15–50 ng/μL and submitted to Affymetrix, Inc. (Santa Clara, CA, United States) for genotyping with the WagRhSNP 68K Axiom SNP array (Koning-Boucoiran et al., 2015). The raw genotypic data acquired from Affymetrix was scored and visually checked using the R packages ‘FitTetra’ (v 1.0; Voorrips et al., 2011) and ‘SNPolisher’ (v 1.5.2; Affymetrix, Inc.) using the default parameters. Each marker on the WagRhSNP 68K Axiom array is represented by two probes (Koning-Boucoiran et al., 2015). Genotypes derived from each replicate were compared. Missing data was imputed from the replicate, when needed. If genotype calls for an individual were different between the two replications the call was converted to a missing score. Markers with greater than 7.5% missing data were removed. Each Axiom marker was assigned an expected cross combination based on the parental genotypes. Offspring genotypes were then checked to determine if any individuals were the result of an unforeseen crossing event, accounting for random and preferential pairing, by checking to see if unexpected allele configurations were present in any of the progeny. Individuals where more than 2.5% of the alleles would not be expected from a ‘Morden Blush’ × Brite Eyes^TM^ cross were considered the result of an unexpected crossing event and would be removed from the analysis.

Prior to mapping, the markers were filtered based on expected segregation ratios for the parental cross accounting for random and preferential pairing, as required for a segmented allopolyploid organism (Bourke et al., 2017). Markers which did not fit expected segregation ratios for either allotetraploid or autotetraploid inheritance (α = 0.01) were removed from analysis. Mapping was conducted using the R package ‘polymapR’ (v 1.0.13; Bourke et al., 2018). Chromosomal linkage groups were established via the “cluster_SN_markers” command using the simplex × nulliplex markers at a LOD of 4. Linkage group homoeologs were identified using duplex × nulliplex markers and the “bridgeHomologues” and “cluster_per_LG” commands. Following homoeolog identification, the remaining segregating marker classes (e.g., simplex × simplex, duplex × duplex, and simplex × duplex) were assigned and mapped using the three-dimensional multi-dimensional scaling algorithm (Preedy and Hackett, 2016) and Kosambi’s mapping function (Kosambi, 1943). Maps were constructed for each homoeolog for both parents and an integrated consensus map was constructed using all homoeologs from both parents. Finally, each linkage group was tested using the “test_prefpairing” command to determine if any genotype dependent preferential pairing occurred that could affect the resulting maps (Bourke et al., 2017).

## Results

### Phenotypic Evaluation

The phenotypic evaluation of the parents revealed Brite Eyes^TM^ was resistant to all of the isolates evaluated except for BEP (race 12; Table 1). ‘Morden Blush’ was susceptible to all of the isolates evaluated (Table 1). As a result, the isolates 2402 E1, GVH, ACT, IGWA, KOMN, CW1, and PAP, which represented races 2, 3, 8, 9, 10, 11, and 13 respectively, were chosen to evaluate the 94 individuals in the population (Whitaker et al., 2010b; D. Zlesak, personal communication). The individuals were either resistant or susceptible to all isolates tested. The progeny segregated 44 resistant to 50 susceptible which fit a 1:1 segregation ratio (χ^2^ = 0.3830, *P* = 0.5360), suggesting the resistance present in Brite Eyes^TM^ is controlled by a single major effect gene. As such, the phenotype was converted to a marker where resistance was scored as a 1 and susceptibility was scored as a 0.

### Linkage Mapping

**FIGURE 1:**
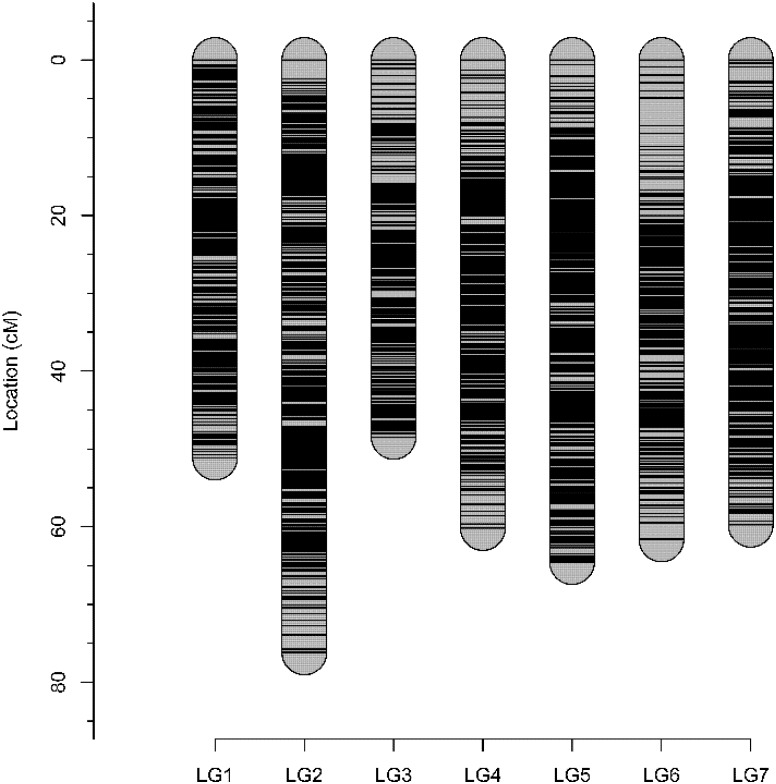
Visual representation of the ‘Morden Blush’ × Brite Eyes^TM^ integrated consensus map.

Genetic analysis identified all 94 individuals to be offspring of both ‘Morden Blush’ and Brite Eyes^TM^ and none were removed for mapping. A total of 28,020 markers were segregating and scored by FitTetra. After filtering based on missing data and tetraploid segregation ratios, 13,682 SNP markers remained. These markers and the phenotype were used for mapping with polymapR. Genomic linkage maps were produced for Brite Eyes^TM^ and ‘Morden Blush’ and chromosomal linkage groups were assigned based on the physical assemblies (Supplementary Tables S1, S2; Hibrand Saint-Oyant et al., 2018; Raymond et al., 2018). The ‘Morden Blush’ map consisted of 6,919 markers distributed over 25 linkage groups and had a total length of 1,371.71 cM. Linkage groups for chromosome 1 homoeolog 4 and chromosome 7 homoeologs 3 and 4 were missing. The markers were distributed into 2,889 bins and the map had a density of 2.11 unique bins/cM. The Brite Eyes^TM^ map consisted of 6,731 markers distributed over 28 linkage groups and had a total length of 1,555.49 cM. Each linkage group represented a different chromosome homoeolog. The markers were distributed into 2,935 bins and the map had a density of 1.89 unique bins/cM. Genotype-dependent preferential pairing was not observed for ‘Morden Blush’ or Brite Eyes^TM^. Additionally, a high degree of collinearity was observed between the genetic positions and the physical positions of the markers on the *Rosa chinensis* genome assemblies (Supplementary Tables S1, S2; Hibrand Saint-Oyant et al., 2018; Raymond et al., 2018). The integrated consensus map consisted of 10,835 markers mapped into 3,929 unique bins (Figure 1 and Supplementary Table S3). The integrated map had a total length of 421.92 cM and a density of 9.31 unique bins/cM. The quality of the integrated linkage map was high and each linkage group had a weighted root mean square error of at most 0.241 (Supplementary Figures S1–S7).

**FIGURE 2:**
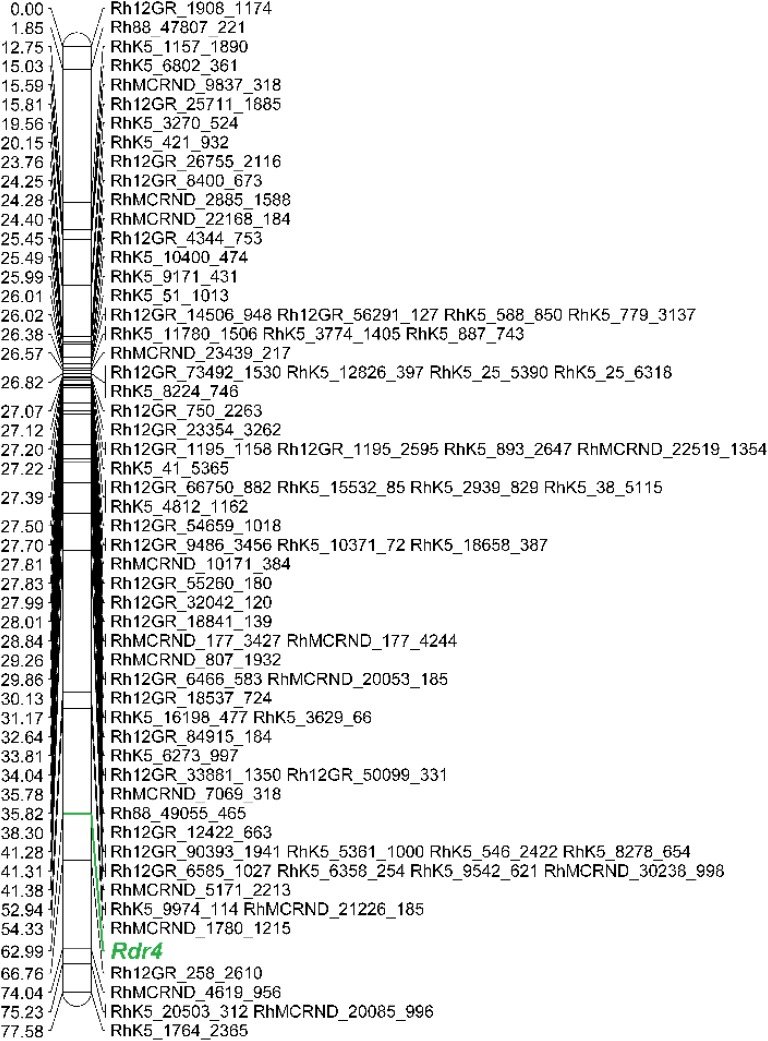
The Brite Eyes^TM^ chromosome 5 homoeolog 4 map. *Rdr4* is displayed in green. This figure was produced using MapChart (v 2.3; Voorrips, 2002).

The black spot resistance present in Brite Eyes^TM^ (*Rdr4*) mapped to the long arm of chromosome 5 homoeolog 4 (Figure 2). The region is 12.43 cM and is delimited proximally by the marker RhMCRND_1780_1215 and distally by Rh12GR_258_2610. Physically this is an 8.37 Mb region when mapping the markers to the Hibrand Saint-Oyant et al. (2018) *R. chinensis* assembly. For the Raymond et al. (2018) *R. chinensis* assembly, this region is larger (9.29 Mb). Gene annotations from each assembly was obtained for the *Rdr4* region and gene function was assessed. Using the Hibrand Saint-Oyant et al. (2018) assembly region, 640 genes were identified and 45 appear to have functions associated with disease resistance (Table 2). A different number of genes (547 genes out of which 52 were associated with disease resistance) were identified in that region when using the Raymond et al. (2018) genome assembly (Table 2). The discrepancy in the number of genes identified can be attributed to the assembly or differences in the methods used to annotate the genes.

**Table 2:** Summary of the resistance gene families identified in the *Rdr4* region for the Hibrand Saint-Oyant et al. (2018) and Raymond et al. (2018) *R. chinensis* genome assemblies.

Gene Family	Hibrand Saint-Oyant et al. (2018) assembly	Raymond et al. (2018) assembly
NB-LRR	9	17
Serine/threonine protein kinase	24	20
Receptor-like kinases	4	11
ABC transporters	8	4
Total # resistance associated genes	45	52

## Discussion

Resistance to black spot is an important trait in roses desired by many home gardeners. The disease resistance present in Brite Eyes^TM^ was a key selling point when the variety was first released (Radler, 2006). The resistance displayed by Brite Eyes^TM^ was also a key trait for selecting it for evaluation in the Earth-Kind^®^ rose trials (Zlesak et al., 2010). Resistance to *D. rosae* races 3, 8, and 9 was assessed during the Earth-Kind^®^ trials, and Brite Eyes^TM^ was found to have resistance to all three races (Zlesak et al., 2010). Identical responses for these races were observed during the present study (Table 1). Further evaluation with additional races revealed Brite Eyes^TM^ was resistant to all races tested except for race 12 (Table 1). This is not unexpected as the race 12 isolate BEP was initially isolated from a Brite Eyes^TM^ plant growing in West Grove, PA (D. Zlesak, personal communication). Further phenotypic observations of the ‘Morden Blush’ × Brite Eyes^TM^ F_1_ population demonstrated that the resistance present in Brite Eyes^TM^ is mediated by a single gene that provides broad resistance. It is unknown which parent contributed resistance to Brite Eyes^TM^ [‘RADtee’ × ‘AUScot’ (synonym Abraham Darby^TM^); Radler, 2006] as these cultivars have not been included in any black spot trials. The black spot resistance gene *Rdr1*, which is hypothesized to be a TIR-NB-LRR, has also been shown to provide a broad level of resistance (Menz et al., 2017). Unlike the resistance present in Brite Eyes^TM^, *Rdr1* does not provide resistance to race 6 isolates of *D. rosae* (Menz et al., 2017).

Three maps were generated, consisting of two parental maps and an integrated consensus map (Supplementary Material 1). The integrated consensus map had a total length of 421.92 cM. This map length is considerably shorter than the integrated consensus map (573.66 cM) generated by Bourke et al. (2017). Despite the difference in length the integrated consensus map generated in this study is thought to be of high quality as the weighted root mean square error for each linkage group was low (Supplementary Material 2), the markers covered most of the physical space for the two *R. chinensis* assemblies, and a high level of collinearity was observed between the map and each *R. chinensis* physical assembly (Supplementary Tables S1–S3; Hibrand Saint-Oyant et al., 2018; Raymond et al., 2018). The weighted root mean square error describes the estimated error when modeling the pairwise estimates of recombination frequency versus the multi-point estimate of the recombination frequency. A high weighted root mean square error would indicate discrepancies between homoeologs and would result in a poor integrated map with inflated distances. There was some abnormality observed for the integrated consensus map for the chromosome 4 linkage group (Supplementary Material 2). This is illustrated in the protrusion in the LOD vs. δ(r) plot and can also be visualized in the recombination frequency plot. These discrepancies can be explained by marker rearrangements between the homoeologs. Such a rearrangement could be expected considering ‘Morden Blush’ and Brite Eyes^TM^ are hybrids and their ancestry may consist of different *Rosa* species that could have rearrangements between homoeologs within the same cultivar or between cultivars.

The ‘Morden Blush’ parental map consisted of 25 linkage groups, each representing a chromosome. Homoeologous linkage groups were not identified for chromosome 1 homoeolog 4 and chromosome 7 homoeologs 3 and 4. During the mapping process chromosomal linkage groups were first established using simplex × nulliplex markers and homoeologous linkage groups were then established using duplex × nulliplex markers. A lack of simplex × nulliplex markers for these chromosomal linkage groups would result in failure to produce linkage maps for these homoeologous linkage groups with unique markers. This problem would be further intensified if the simplex × nulliplex markers present for this linkage group are sufficiently distant. Conversely, linkage groups would not be established if the simplex × nulliplex markers available for these homoeologs did not have linkage with the bridging duplex × nulliplex or simplex × simplex markers. The number of simplex × nulliplex markers present for a sample would also be affected by any ascertainment bias in the array. Ascertainment biases are a common occurrence in microarray-based genotyping that can be mitigated by using a highly diverse set of individuals during the SNP discovery process (Albrechtsen et al., 2010).

The Brite Eyes^TM^ parental map consisted of 28 linkages groups each representing one of the 28 chromosomes. The single gene resistance present in Brite Eyes^TM^ (*Rdr4*) mapped to the fourth homoeolog of chromosome 5. The *Rdr4* region is within a 12.43 cM region and the nearest marker is 3.77 cM away. Marker density in this region is lower than other regions of the chromosome. Markers that mapped to the *Rdr4* physical region were found to be monomorphic in both parents, map to one of the ‘Morden Blush’ chromosome 5 homoeologous linkage groups, or map to one of the other Brite Eyes^TM^ chromosome 5 homoeologous linkage groups. Considering the potential for ascertainment bias within the WagRhSNP 68K Axiom array, it is possible that there are polymorphisms within the *Rdr4* region that have not been mapped (Albrechtsen et al., 2010). Further polymorphism discovery will be needed to improve marker density within the region.

To date, only three black spot resistance genes; *Rdr1, Rdr2*, and *Rdr3*; have been reported (von Malek and Debener, 1998; Hattendorf et al., 2004; Whitaker et al., 2010a; Menz et al., 2017). Many additional unidentified genes are expected to exist based on the host-pathogen interactions observed in the differential set (Whitaker et al., 2010b). Both *Rdr1* and *Rdr2* map to chromosome 1 indicating that *Rdr4* is different from either of these genes. *Rdr3* was originally identified in ‘George Vancouver’ and its chromosomal location is unknown (Whitaker et al., 2010a). ‘George Vancouver’ was evaluated during the Earth-Kind^®^ black spot trials and was found to be susceptible to race 3 and 9 *D. rosae* isolates (Zlesak et al., 2010). Phenotyping the ‘Morden Blush’ × Brite Eyes^TM^ progeny demonstrated that *Rdr4* confers resistance to both races 3 and 9. As such, *Rdr4* is a novel black spot resistance gene or an allele of *Rdr3*. Mapping *Rdr3* will be needed to confirm the novelty of *Rdr4*. Future work will focus on further characterizing *Rdr4* and developing a DNA test for marker assisted breeding. Despite its broad level of resistance, we still propose that all future breeding work should strive to deploy *Rdr4* in conjunction with other sources of black spot resistance to maintain its efficacy.

## Data Availability

Data analyzed for this study are available upon reasonable request. Requests for mapping data should be directed to Nahla Bassil (Nahla.Bassil@ars.usda.gov). Requests for germplasm, as available, should be directed to Stan Hokanson (hokan017@umn.edu). Germplasm may require material transfer agreements as subject to institutional policy.

## Author Contributions

JZ conducted the analysis and wrote the manuscript. DZ, MH, and SH conceived the experiments, conducted the phenotypic assays, and maintained the plants. JB provided laboratory space to conduct the phenotypic assays. NB conceived the experiments and wrote the manuscript. All authors reviewed and edited the final manuscript.

## Conflict of Interest Statement

The authors declare that the research was conducted in the absence of any commercial or financial relationships that could be construed as a potential conflict of interest.
