# Supplementary material for: Mapping a Novel Black Spot Resistance Locus in the Climbing Rose Brite Eyes™ (‘RADbrite’)
Source: Front Plant Sci. 2018 Nov 26;9:1730. doi: 10.3389/fpls.2018.01730 (PMC6275305; doi:10.3389/fpls.2018.01730)
Supplement: Supplementary file 1 [file Data_Sheet_1.pdf]

Mapping a Novel Black Spot Resistance Locus in the Rose Cultivar Brite Eyes^{TM} ('RADbrite')

Jason D. Zurn[1], David C. Zlesak[2], Matthew Holen[3], James Bradeen[4], Stan C. Hokanson[3], Nahla V. Bassil[1]

[1] USDA-ARS National Clonal Germplasm Repository, Corvallis, OR, U.S.A.

[2] Department of Plant and Earth Science, University of Wisconsin River Falls, River Falls, WI, U.S.A.

[3] Department of Horticulture, University of Minnesota, St. Paul, MN, U.S.A.

[4] Department of Plant Pathology, University of Minnesota, St. Paul, MN, U.S.A.

*Corresponding Email: Nahla.Bassil@ars.usda.gov Tel: 541-738-4214 Fax: 541-738-4205

Supplementary Figures S1-S7. The diagnostic plots produced by ‘polymapR’ (Bourke et al., 2018) to assess the integrated consensus map quality. **A)** Plot of the pairwise estimates of recombination frequency and the effective estimate of recombination frequency based on the multi-point estimate. High quality integrated linkage groups have a low weighted root mean square error (RMSE). **B)** Heat map displaying the comparison between a marker’s map position and the recombination frequency estimates. **C)** Heat map displaying the comparison between a marker’s map position and the LOD estimates relative to other markers with the linkage group.

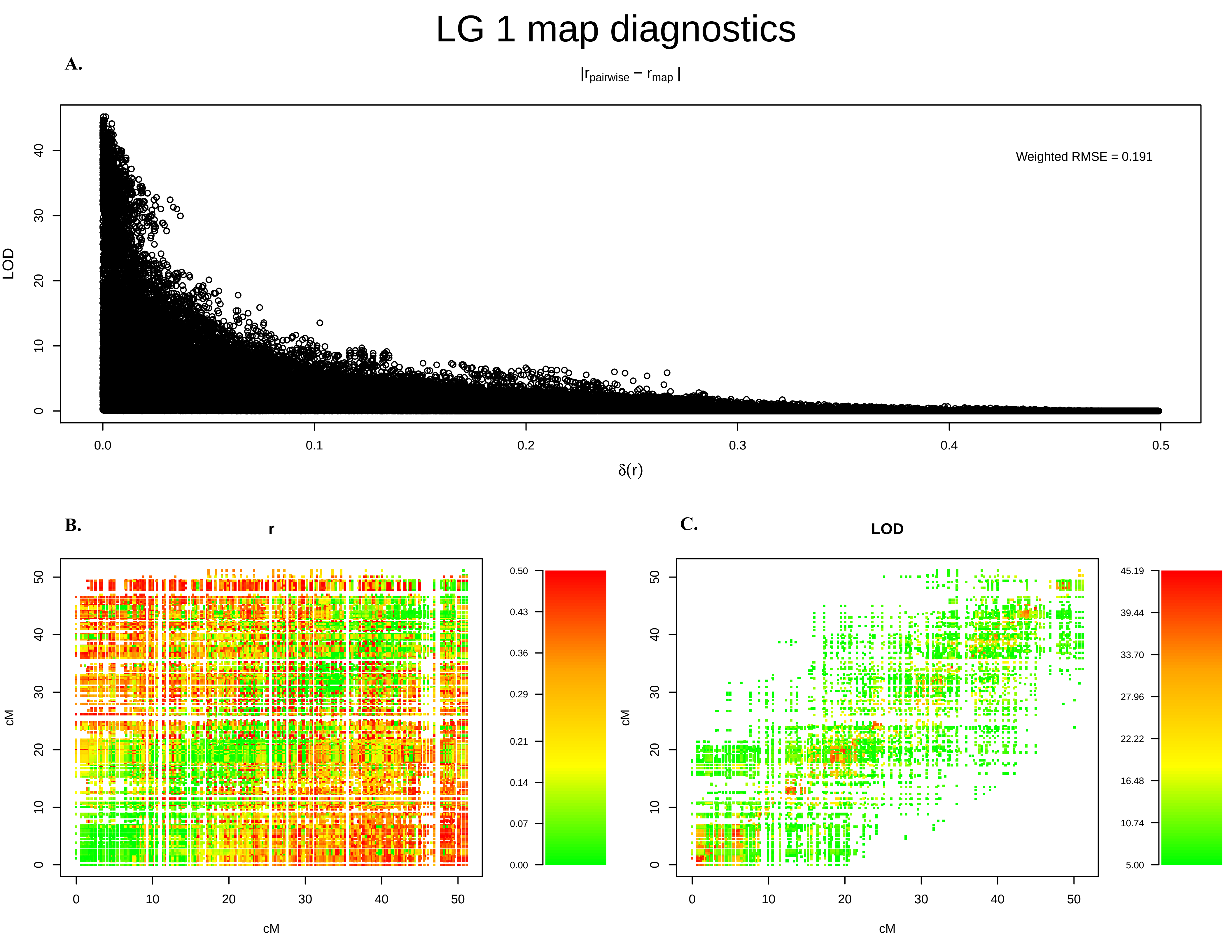
LG 1 map diagnostics
A.
|r_pairwise − r_map |
Weighted RMSE = 0.191
LOD
40
30
20
10
0
0.0
0.1
0.2
0.3
0.4
0.5
δ(r)
B.
r
cM
50
40
30
20
10
0
0
10
20
30
40
50
cM
0.50
0.43
0.36
0.29
0.21
0.14
0.07
0.00
C.
LOD
cM
50
40
30
20
10
0
0
10
20
30
40
50
cM
45.19
39.44
33.70
27.96
22.22
16.48
10.74
5.00

LG 2 map diagnostics

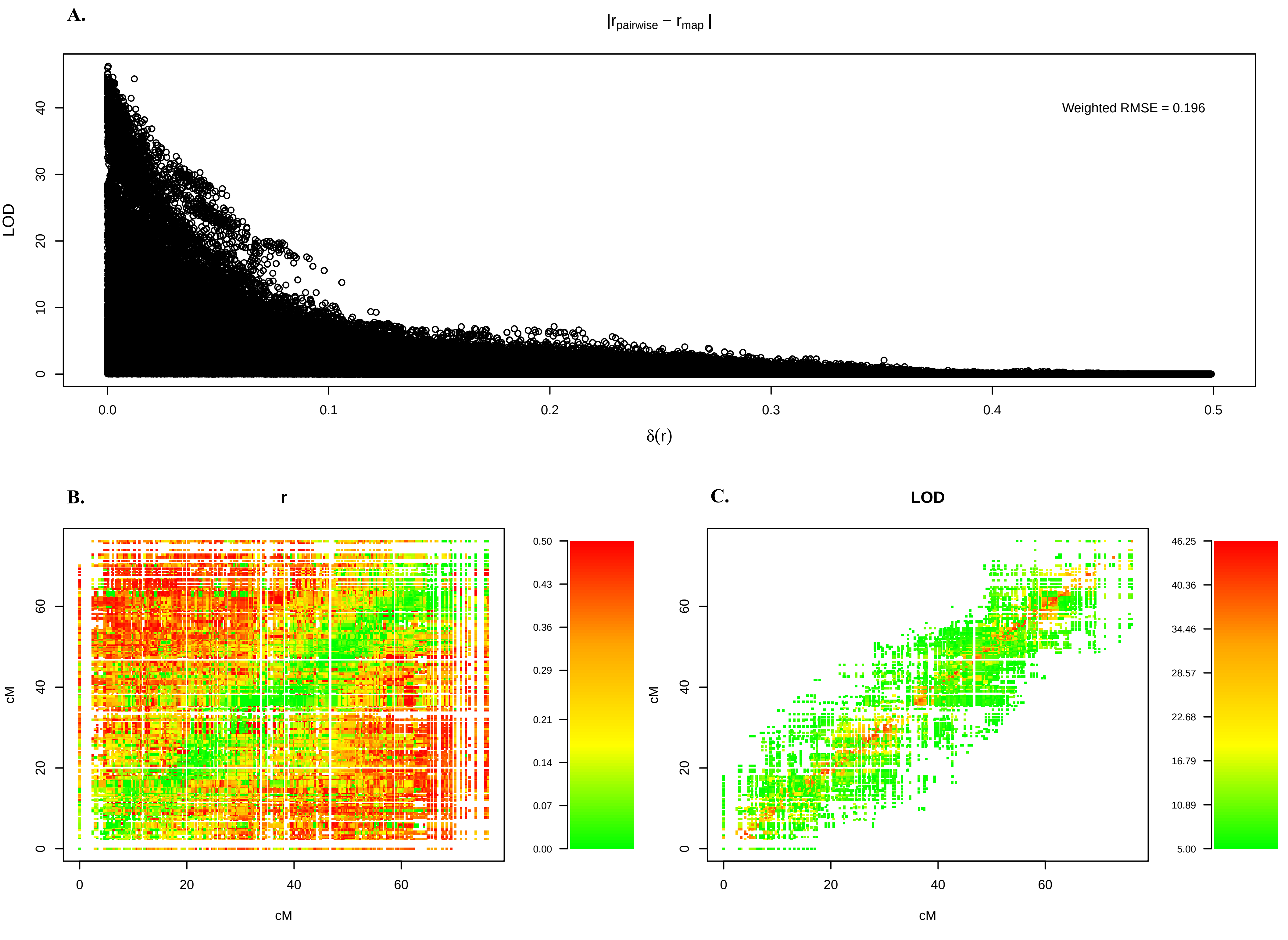

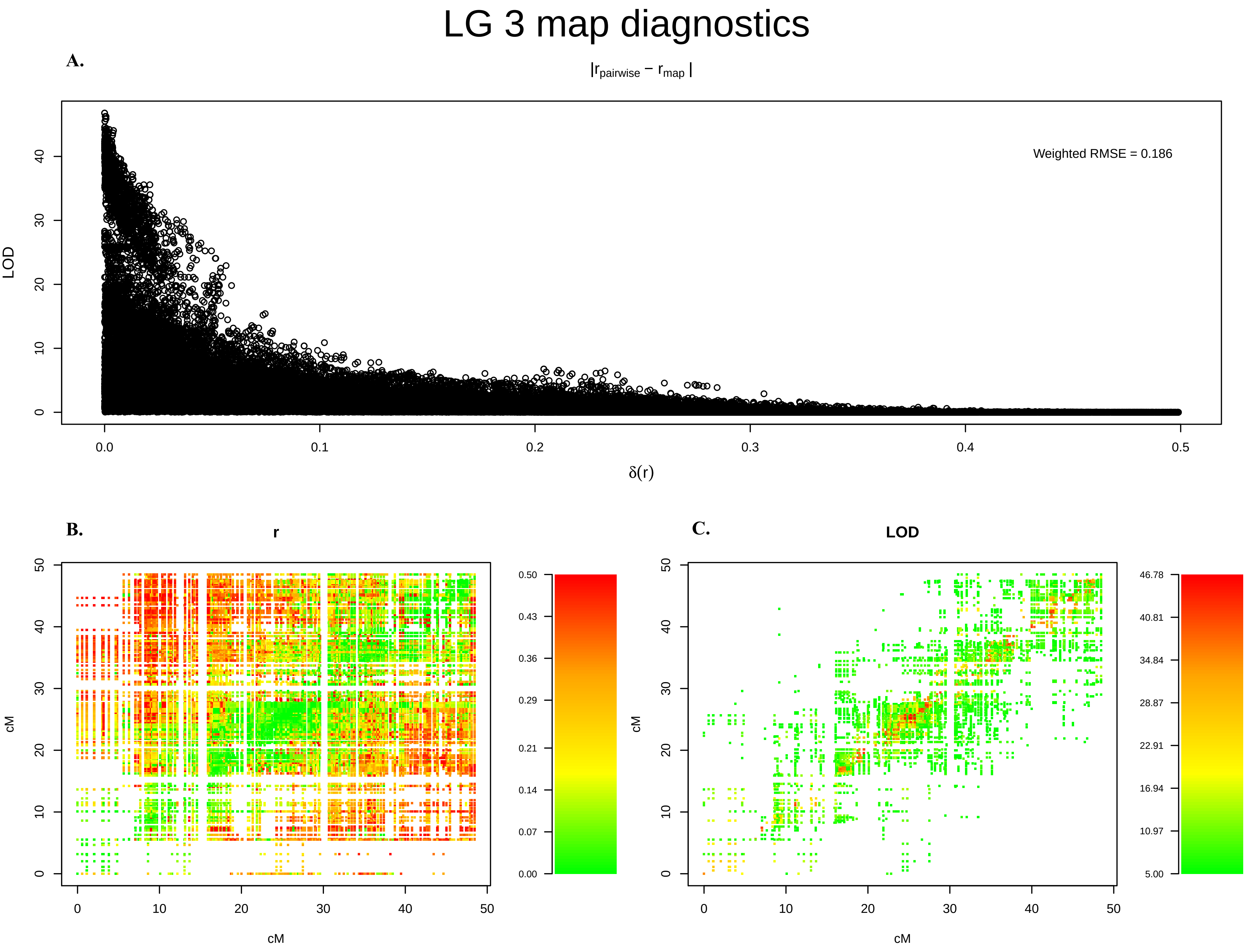
LG 3 map diagnostics
A.
|r_pairwise − r_map |
Weighted RMSE = 0.186
LOD
δ(r)
B.
r
cM
cM
C.
LOD
cM
cM
0.50
0.43
0.36
0.29
0.21
0.14
0.07
0.00
46.78
40.81
34.84
28.87
22.91
16.94
10.97
5.00

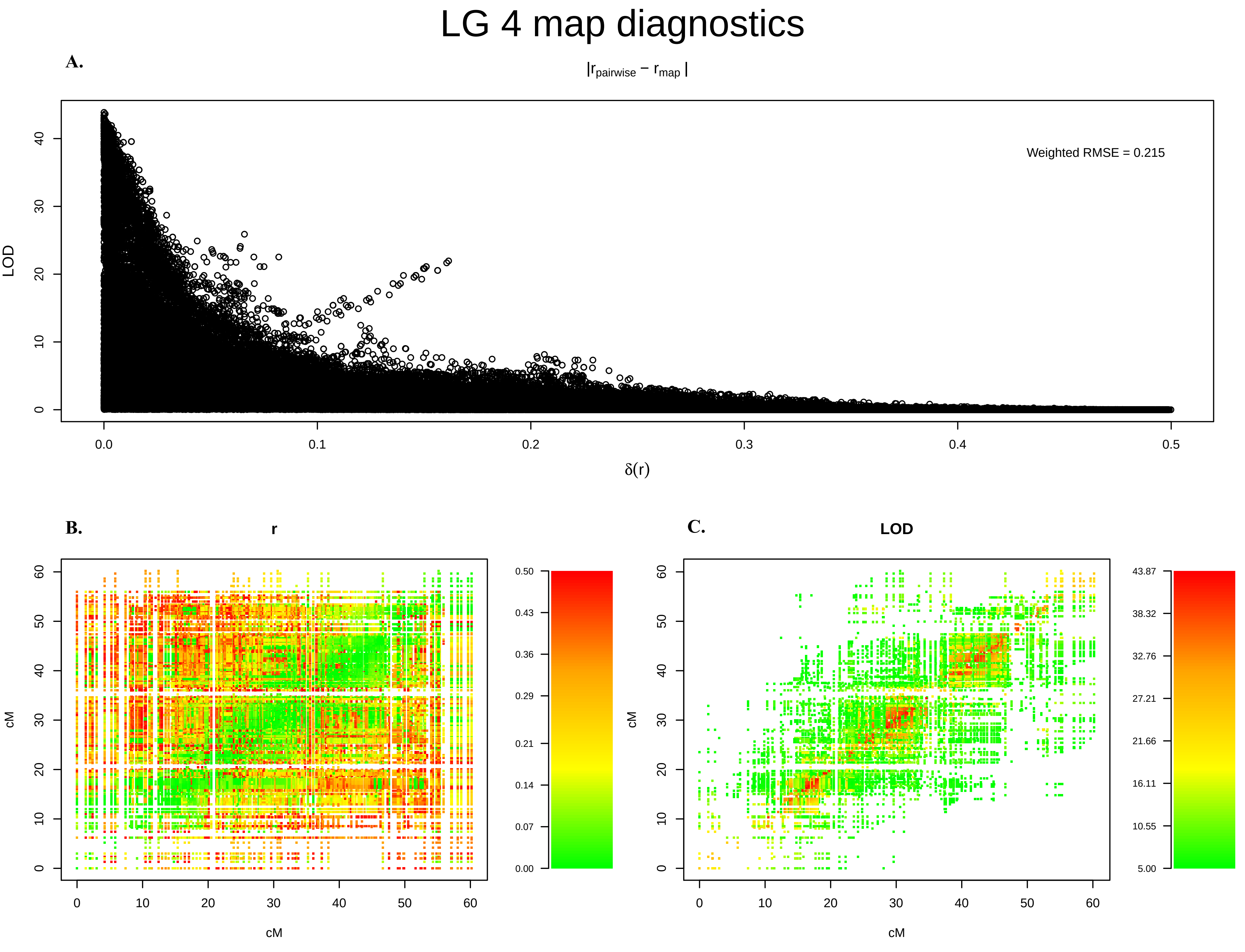
LG 4 map diagnostics
A.
|rpairwise − rmap |
Weighted RMSE = 0.215
LOD
δ(r)
0.0
0.1
0.2
0.3
0.4
0.5
0
10
20
30
40
B.
r
C.
LOD
cM
0.50
0.43
0.36
0.29
0.21
0.14
0.07
0.00
43.87
38.32
32.76
27.21
21.66
16.11
10.55
5.00

LG 5 map diagnostics

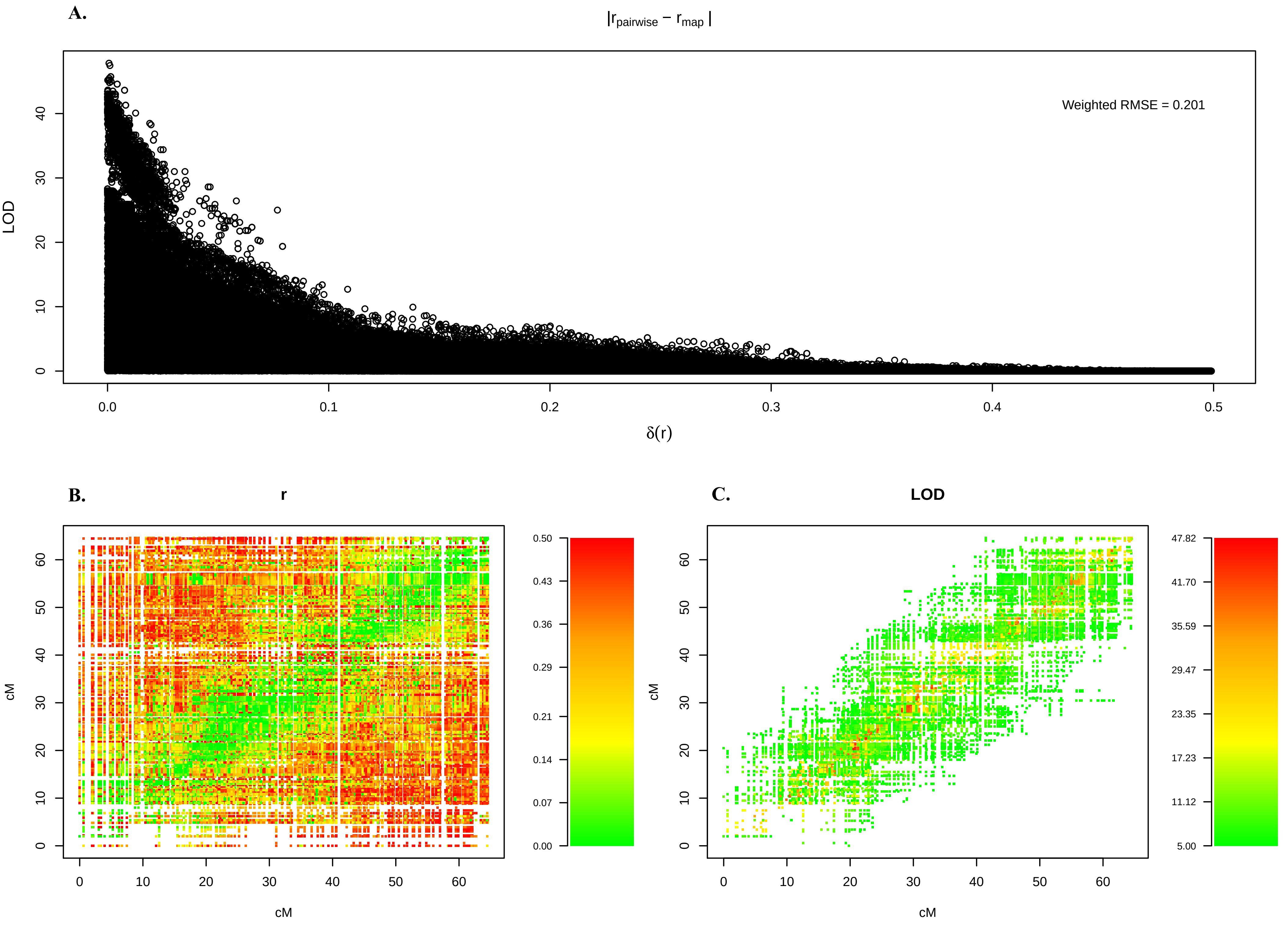

LG 6 map diagnostics

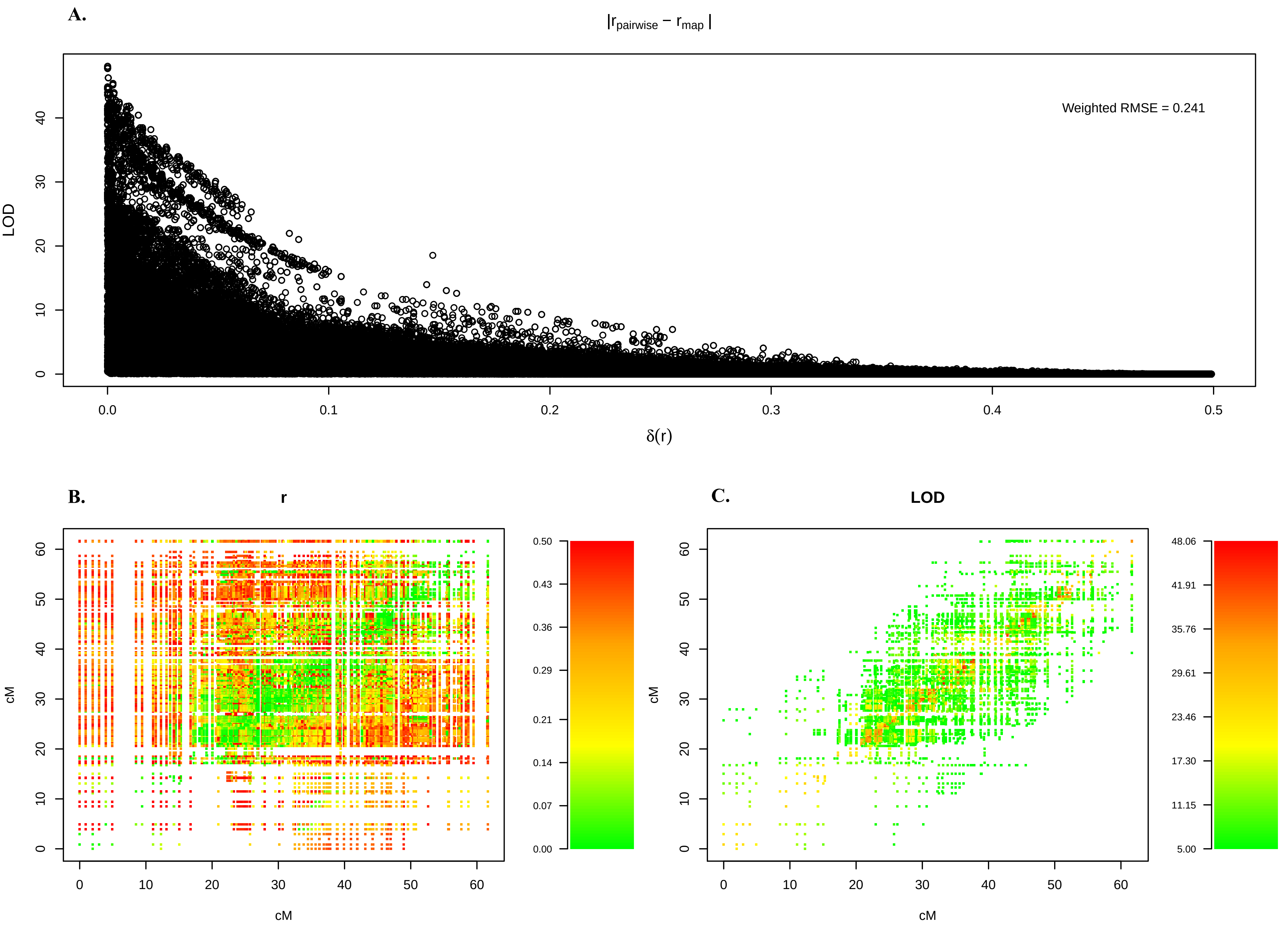

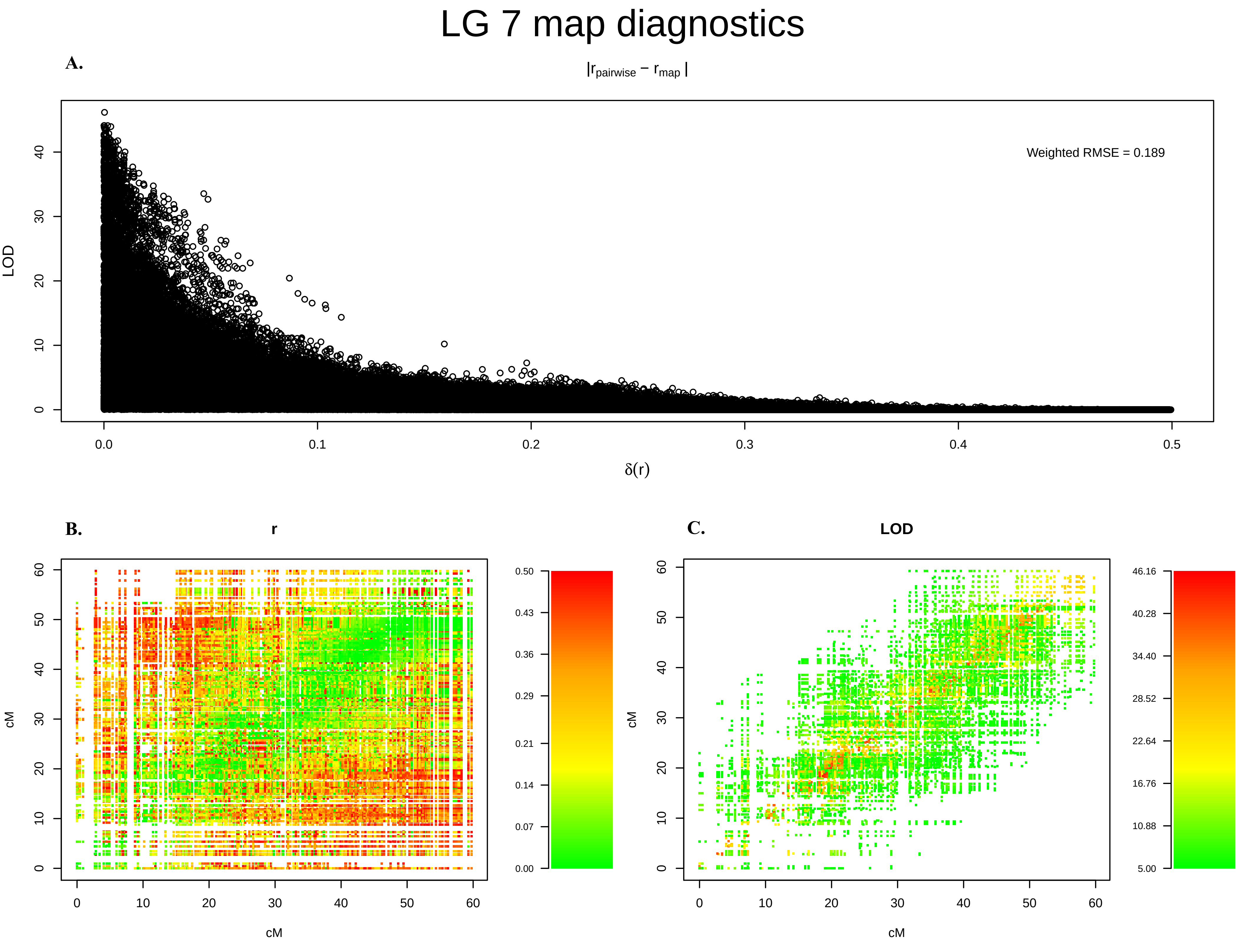
LG 7 map diagnostics
A.
|rpairwise − rmap |
Weighted RMSE = 0.189
LOD
0
10
20
30
40
0.0
0.1
0.2
0.3
0.4
0.5
δ(r)
B.
r
cM
0
10
20
30
40
50
60
0.50
0.43
0.36
0.29
0.21
0.14
0.07
0.00
C.
LOD
46.16
40.28
34.40
28.52
22.64
16.76
10.88
5.00
